# Gut microbiota and fecal metabolites in captive and wild North China leopard (*Panthera pardus japonensis*) by comparsion using 16 s rRNA gene sequencing and LC/MS-based metabolomics

**DOI:** 10.1186/s12917-020-02583-1

**Published:** 2020-09-29

**Authors:** Yan Hua, Heqin Cao, Jiao Wang, Fengping He, Guangshun Jiang

**Affiliations:** 1 Feline Research Center of National Forestry and Grassland Administration, College of Wildlife and Natural Protected Area, Northeast Forestry University, 150040 Harbin, China; 2 Guangdong Provincial Key Laboratory of Silviculture, Protection and Utilization, Guangdong Academy of Forestry, 510520 Guangzhou, China; 3 College of Veterinary Medicine, Yunnan Agricultural University, 650201 Kunming, China

**Keywords:** Fecal metabolites, gut microbiota, North China leopard, 16S rRNA gene sequencing, metabolomics

## Abstract

**Background:**

**G**ut microbes significantly contribute to nutrient digestion and absorption, intestinal health and immunity, and are essential for the survival and environmental adaptation of wild animals. However, there are few studies on the gut microbiota of captive and wild North China leopard (*Panthera pardus japonensis*).

**Results:**

A total of 10 mainly bacterial phyla were identified in the fecal microbiota of North China leopard, *Lachnoclostridium* (*p* = 0.003), *Peptoclostridium* (*p* = 0.005), *Bacteroides* (*p* = 0.008), *Fusobacterium* (*p* = 0.017) and *Collinsella* (*p* = 0.019) were significantly higher than those of wild North China leopard. Distinct differences in the fecal metabolic phenotypes of captive and wild North China leopard were found, such as content of l-methionine, n-acetyl-l-tyrosine, pentadecanoic acid and oleic acid. Differentially abundant gut microbes were associated with fecal metabolites, especially the bacteria in Firmicutes and Bacteroidetes, involved in the metabolism of N-acetyl-L-alanine and D-quinovose.

**Conclusion:**

This study reports for the first time the differences in gut microbiota abundance between captive and wild North China leopard, as well as significant differences in fecal metabolic phenotypes between two groups.

## Background

The North China leopard (*Panthera pardus japonensis*), also called the China leopard, is one of the three native leopard subspecies distributed in China. The North China leopard is only found in the eastern and central parts of China [1]. Due to habitat fragmentation, large-scale forest reduction and poor habitat connectivity, the distribution range and population size of the North China leopard have sharply decreased [2–4], and it has been listed as a national class I key protected animal. It has also been listed as endangered (EN) by the Red List of Biodiversity in China-Vertebrate Volume. China's Red Book of Endangered Animals classified it as Endangered (E); it was upgraded from Vulnerable (VU) in 2008 to Near Threatened (NT) by the IUCN, and has been listed in Appendix I by CITES.

Fortunately, with the strengthening of protection, the North China leopard population shows stable and an increasing trend. However, due to its limited habitat areas and diets, there is an obviously increased risk of attack by diseases, especially intestinal diseases, which threaten the health and life of animals [5]. Gut microbial diversity analyses based on leopard fecal samples should also be considered as important insights on conservation.

Many studies have shown that the gastrointestinal tracts of human beings and animals contains large and complex microbial communities [6], and changes in the gut microbiota have been shown to affect host metabolism and energy homeostasis [7]. 16S rRNA sequencing technology is widely used in the study of the composition and abundance of the human and animal gut microbiota [8, 9]. Researchers have also revealed differences in the composition and structure of the gut microbiota between captive and wild animals, and the stability of the gut microbiota is closely related to the health of the population [10, 11]. The food of carnivores usually contains a large amount of animal tissue, which provides a substrate for intestinal microorganisms to use for fermentation [12, 13]. Specific compositions of the gut microbiota are associated with variations in the host diet and physiological status [14, 15]. Therefore, the study on the gut microbiota diversity of carnivores with different diets and living environments can better reveal the differences in the functional contribution of the gut microbiota.

Furthermore, regarding the relationship between gut microbes and the host fecal metabolic phenotype, non-targeted metabolomics can be used to explore how gut microbes affect metabolic function. Metabolomics analysis can reveal the abnormal metabolism and metabolic pathways involved in complex diseases [16]. Non-targeted fecal metabolome studies have been used to unravel metabolic phenotypic variation associated with gut microflora disturbances during disease occurrence [17].

Facing the wild environments, the wild North China leopard coevolves with its surroundings to help it adapt to the wild environment. In contrast, captive individuals are faced with specific artificial feeding environments and dietary conditions that are different from the wild [18]. Under such conditions, the metabolism and behavior of captive North China leopard may change, thus affecting the composition, structure and function of their gut microbiota. The gut microbiota of captive and wild North China leopard, and the relationship between microbial community and fecal metabolism phenotype, is of great importance for the healthy development of the North China leopard population. In this study, we want to reveal differences in the gut microbiota of captive and wild leopard, and the relationships between the gut microbiota and the fecal metabolic phenotype using an analysis method combining 16S rRNA gene sequencing and LC-MS metabolomics, providing scientific guidance for the protection and breeding of leopard population.

## Results

### Gut microbiota of the North China leopard

Based on 16S rRNA gene sequencing, the fecal microbiota composition of captive and wild North China leopard was analyzed, and 39.01G of high-quality leopard base sequences without adaptor and DNA contamination was obtained.

**Fig. 1:**
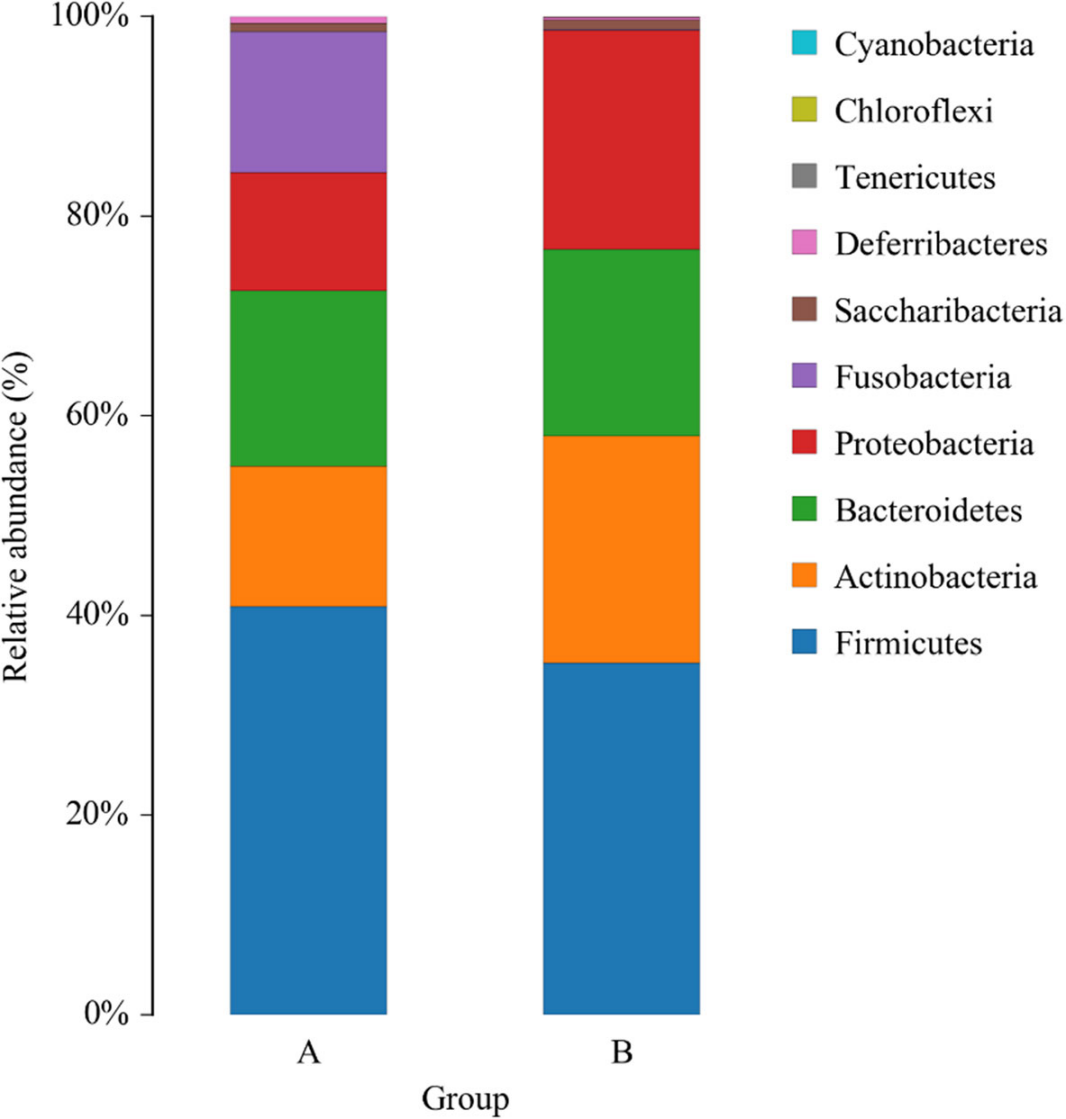
Relative gut microbiota abundance at the phylum level in captive and wild North China leopard .Note: A group is the captive North China leopard, and B group is the wild North China leopard

A total of 10 bacterial phyla were identified in the fecal microbiota 16S rRNA sequencing (Fig. 1). Firmicutes (41%), Bacteroidetes (17.8%), Fusobacteria (14.3%), Actinobacteria (13.8%), Proteobacteria (11.5%) and Saccharibacteria (8.22%) were the main bacteria components of captive North China leopard. In the wild individuals, Firmicutes (34.3%), Actinobacteria (23.4%), Proteobacteria (21.8%), Bacteroidetes (19.2%) and Saccharibacteria (8.98%) were the main components. The fecal microbial composition of captive and wild animals was significantly different for Fusobacteria (*p* = 0.036), and the relative abundance was significantly higher than that of the wild leopard, but there was no significant difference at other phylum levels.

**Fig. 2:**
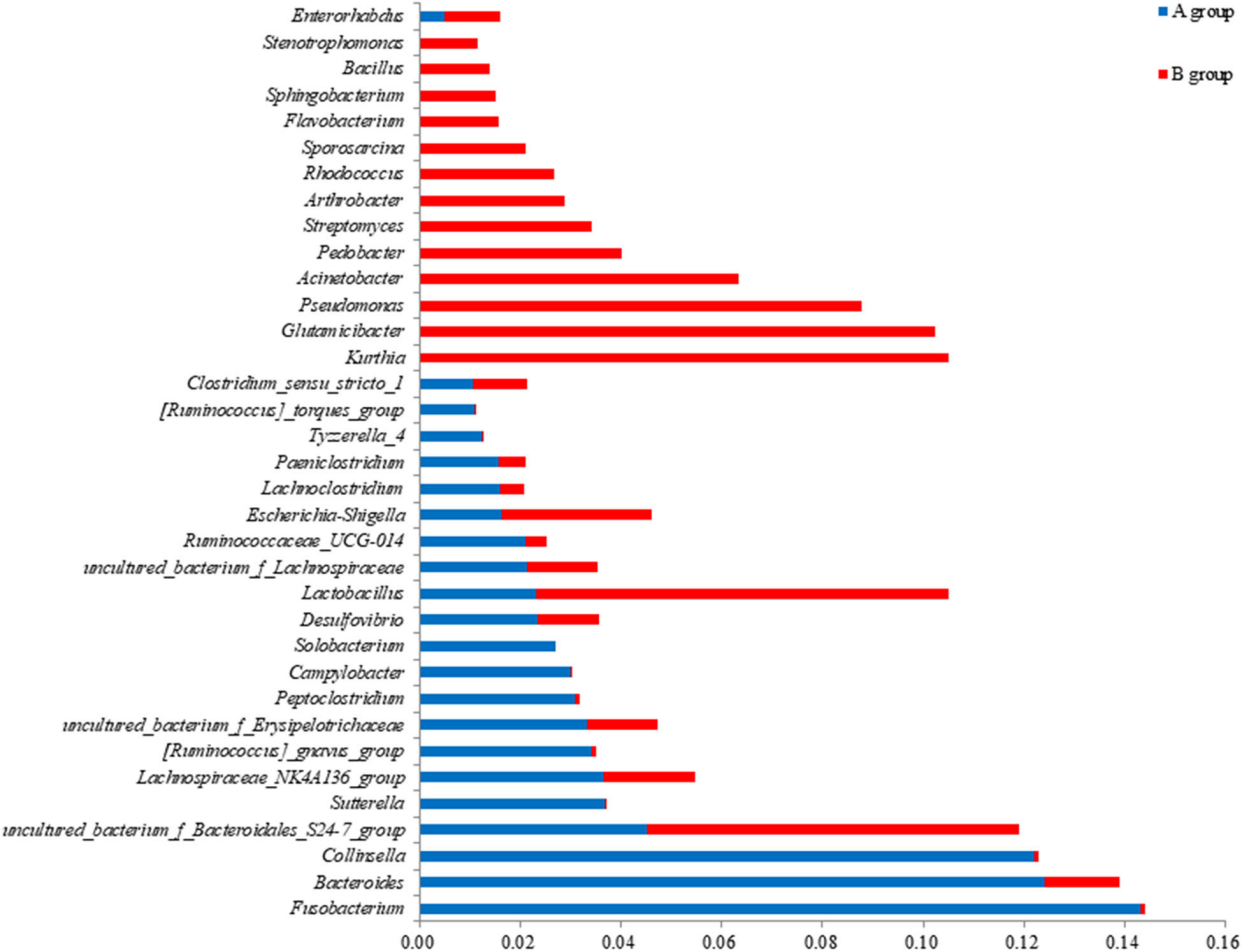
Relative gut microbiota abundance at the genus level in captive and wild North China leopard. Note: A group is the captive North China leopard, and B group is the wild North China leopard

At the genus level, 18 genera showed significant differences between captive and wild leopards (Fig. 2), and the relative abundance of 11 genera, including *Lachnoclostridium* (*p* = 0.003), *Peptoclostridium* (*p* = 0.005), *Bacteroides* (*p* = 0.008), *Fusobacterium* (*p* = 0.017) and *Collinsella* (*p* = 0.019), were significantly higher than those of wild leopards. There were 21 dominant genera in captive leopards and 23 dominant genera in wild North China leopard. *Fusobacterium*, *Bacteroides* and *Collinsella* were the dominant genera in the captive leopards. The wild leopards were completely different, and *Kurthia*, *Glutamicibacter*, *Pseudomonas* and *Lactobacillus* were the dominant genera. There were 9 common dominant genera of both groups, including *Bacteroides*, *Lactobacillus*, *Desulfovibrio*, *Escherichia - Shigella*, *Lachnospiraceae _ NK4A136 _ group*, *bacterium_f_bacteroidales_s24-7_group*, *Uncultured _ bacterium _ f _ Lachnospiraceae*, *uncultured _ bacterium _f _ Erysipelotrichaceae* and *Clostridium_sensu_stricto_1*.

### Fecal metabolic profiling of North China leopard

**Fig. 3:**
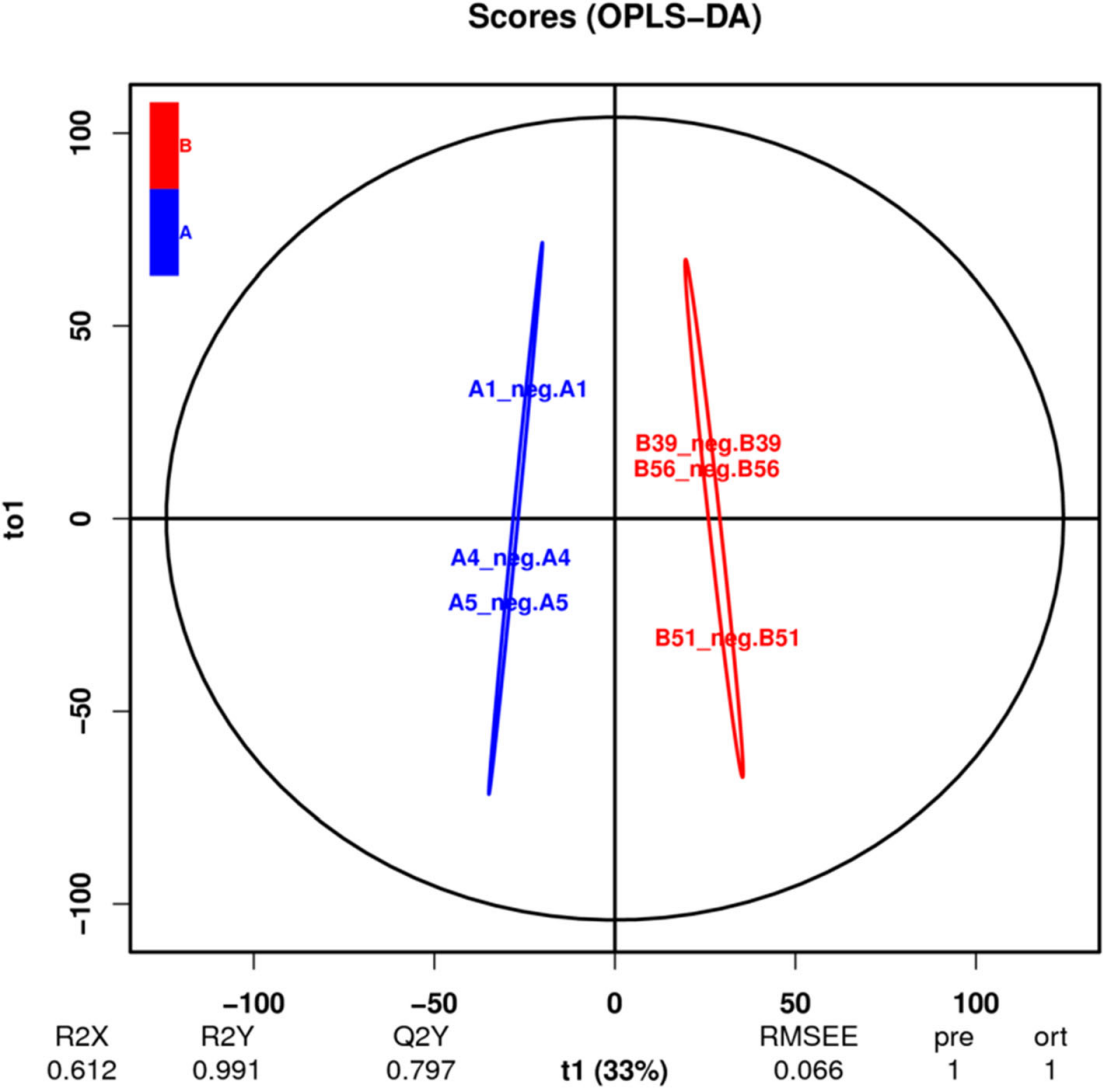
OPLS-DA score of captive and wild North China leopard. Note: A1, A4 and A5 are captive North China leopard, B39, B51 and B56 are wild North China leopard

The fecal metabolic profiles of North China leopard were acquired by LC-MS. In the OPLS-DA model, the metabolic curves were obviously different, and the results showed that there were obvious differences in fecal metabolites between captive and wild leopard (Fig. 3).

**Fig. 4:**
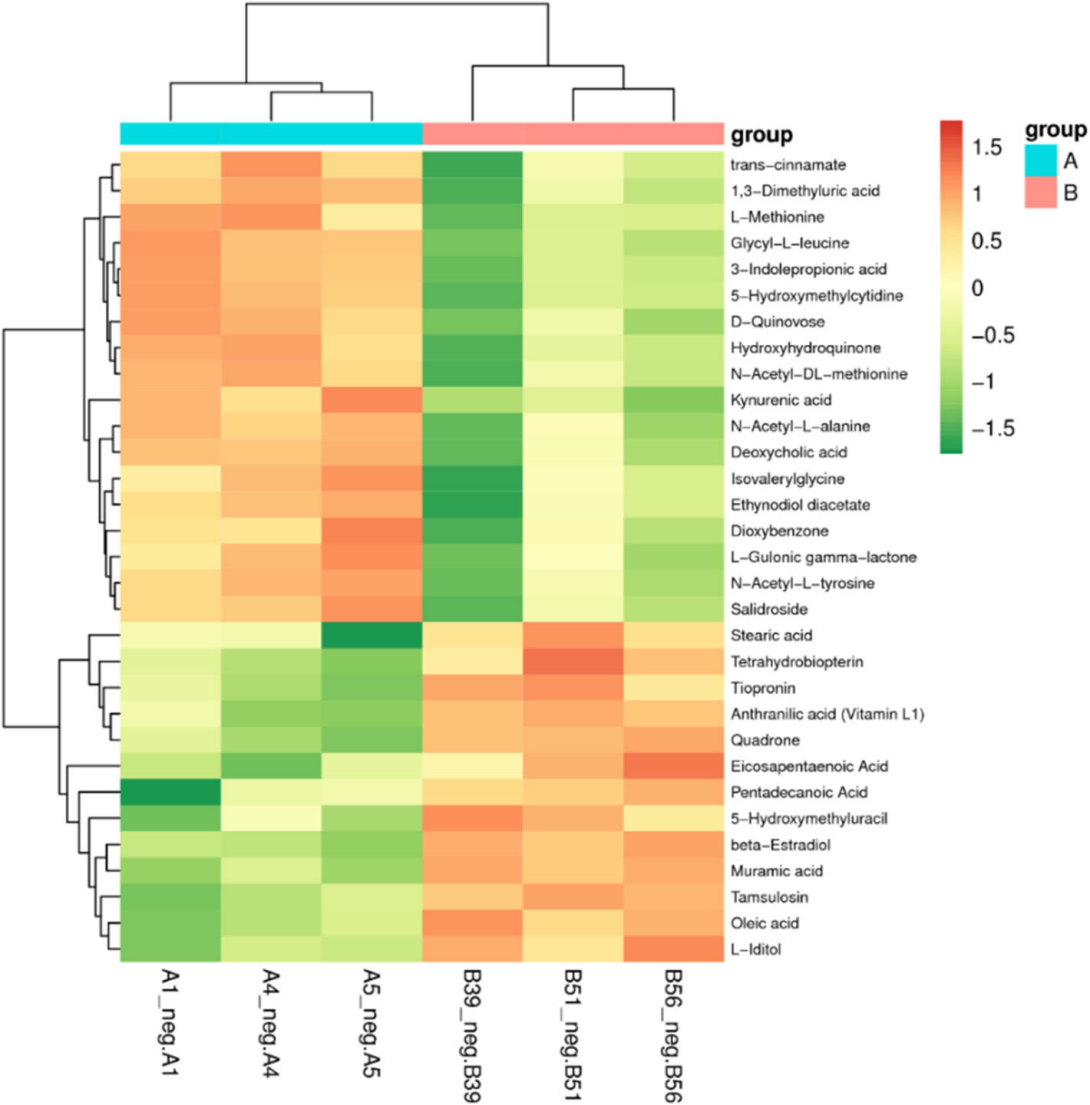
Heat map of metabolites with significant differences based on LC/MS data of fecal samples. Red and green represent higher and lower concentrations of metabolites in captive and wild North China leopard, respectively

The metabolites were identified by MS/MS based on accurate molecular weights and fragment patterns, and by comparison to online databases such as the Human Metabolome Database (HMDB;http://www.hmdb.ca/), Biofluid Metabolites Database (http://metlin.scripps.edu) and Massbank (http://www.massbank.jp/). The results showed that there were a total of 31 metabolites with significant differences between captive and wild North China leopard, and 9 metabolites with extremely significant differences (Fig. 4). The corresponding *P* values and VIP values are shown in Table 1.

**Table 1:** Metabolites differences between captive and wild North China leopard achieved from the analyses of feces samples submitted to liquid chromatography/mass spectrometer (LC-MS) analyses using an Ultra High Performance Liquid Chromatography (UHPLC) system

No.	Metabolites	*P* value	VIP	Regulated
999	N-acetyl-L-alanine	0. 006	1. 602	Up^b^
1155	anthranilic acid (vitamin L1)	0. 004	1. 723	Down^b^
1331	hydroxyhydroquinone	0. 022	1. 595	Up^a^
1474	trans-cinnamate	0. 044	1. 534	Up^a^
1492	L-methionine	0. 049	1. 514	Up^a^
1814	isovalerylglycine	0. 041	1. 517	Up^a^
1945	5-hydroxymethyluracil	0. 022	1. 529	Down^a^
2713	tiopronin	0. 044	1. 610	Down^a^
2850	kynurenic acid	0. 011	1. 630	Up^a^
2856	3-indolepropionic acid	0. 011	1. 640	Up^a^
2869	glycyl-L-leucine	0. 039	1. 571	Up^a^
2925	N-acetyl-DL-methionine	0. 019	1. 617	Up^a^
3103	1, 3-dimethyluric acid	0. 025	1. 659	Up^a^
4329	N-acetyl-L-tyrosine	0. 018	1. 641	Up^a^
4374	D-quinovose	0. 004	1. 603	Up^b^
5127	L-gulonic gamma-lactone	0. 024	1. 521	Up^a^
5361	tetrahydrobiopterin	0. 037	1. 608	Down^a^
5372	pentadecanoic Acid	0. 005	1. 599	Down^b^
5531	dioxybenzone	0. 045	1. 417	Up^a^
5714	quadrone	0. 000	1. 725	Down^b^
6094	5-hydroxymethylcytidine	0. 019	1. 619	Up^a^
7596	oleic acid	0. 032	1. 582	Down^a^
7742	stearic acid	0. 034	1. 542	Down^a^
10198	beta-Estradiol	0. 007	1. 687	Down^b^
10469	salidroside	0. 039	1. 582	Up^a^
10588	eicosapentaenoic Acid	0. 049	1. 501	Down^a^
11683	L-iditol	0. 044	1. 522	Down^a^
13489	ethynodiol diacetate	0. 016	1. 637	Up^a^
13779	tamsulosin	0. 000	1. 707	Down^b^
18005	muramic acid	0. 000	1. 689	Down^b^
24886	deoxycholic acid	0. 006	1. 705	Up^b^

Note: ^a^means significant difference, and ^b^means extremely significant difference, Up means the fecal metabolites of captive North China leopard are higher than those in the wild, Down means the fecal metabolites of wild North China leopard are higher than those in the captive.

A heat map shows the trends of the metabolites of captive and wild leopard(Fig. 4), and a total of 18 metabolites were significantly higher in captive than wild leopard(N-acetyl-L-alanine, hydroxyhydroquinone, trans-cinnamate, L-methionine, isovalerylglycine, kynurenic acid, 3-indolepropionic acid, glycyl-L-leucine, N-acetyl-dl-methionine, 1,3-dimethyluric acid, n-acetyl-l-tyrosine, d-quinovose, l-gulonic gamma-lactone, Dioxybenzone, 5-hydroxymethylcytidine, Salidroside, Ethynodiol diacetate, deoxycholic acid); 13 metabolites were significantly lower than that of wild leopard(vitamin L1, 5-hydroxymethyluracil, Tiopronin, tetrahydrobiopterin, pentadecanoic acid, quadrone, oleic acid, stearic acid, beta-estradiol, eicosapentaenoic acid, l-iditol, tamsulosin, muramic acid).

### 2.3 Correlation of gut microbiota with fecal metabolic phenotype of North China leopard

**Fig. 5:**
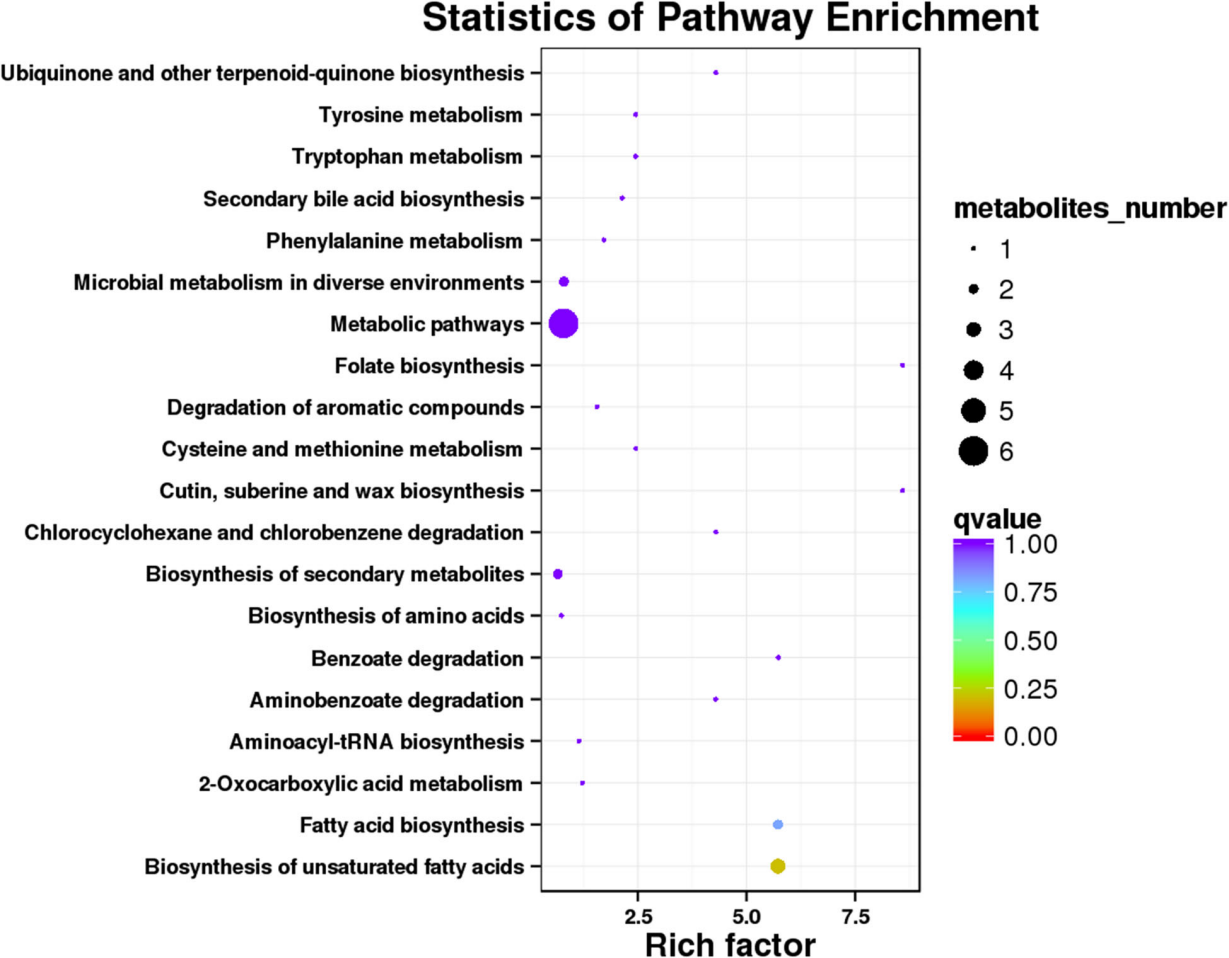
Statistics of different metabolites on pathway enrichment. Note: The rich factor is the ratio of the proportion of different metabolites in the pathway and the proportion of all metabolites in the pathway. The larger the value is, the greater the enrichment degree is. Qvalue is Pvalue after multiple hypothesis test correction, and the closer it is to 0, the more significant the enrichment is. The size of the points in the figure represents the number of metabolites enriched in the corresponding pathway

To explore the correlation with significant differences in fecal metabolites and microorganisms between captive and wild leopards, we used the MetPA database, which is a part of MetaboAnalyst (www.metaboanalys.ca), and the Kyoto Encyclopedia of Genes and Genomes (KEGG) to analyze the pathway of the metabolites that differed between these two leopard groups (Fig. 5). The results showed that the relative abundances of KEGG first-order metabolic pathways such as Metabolism, Genetic Information Processing and Human Diseases were the highest among the gut microbiota of both captive and wild leopards. The metabolites enriched in Biosynthesis of unsaturated fatty acids were significantly different, and the amount of different metabolites enriched in the metabolic pathways was the largest.

**Fig. 6:**
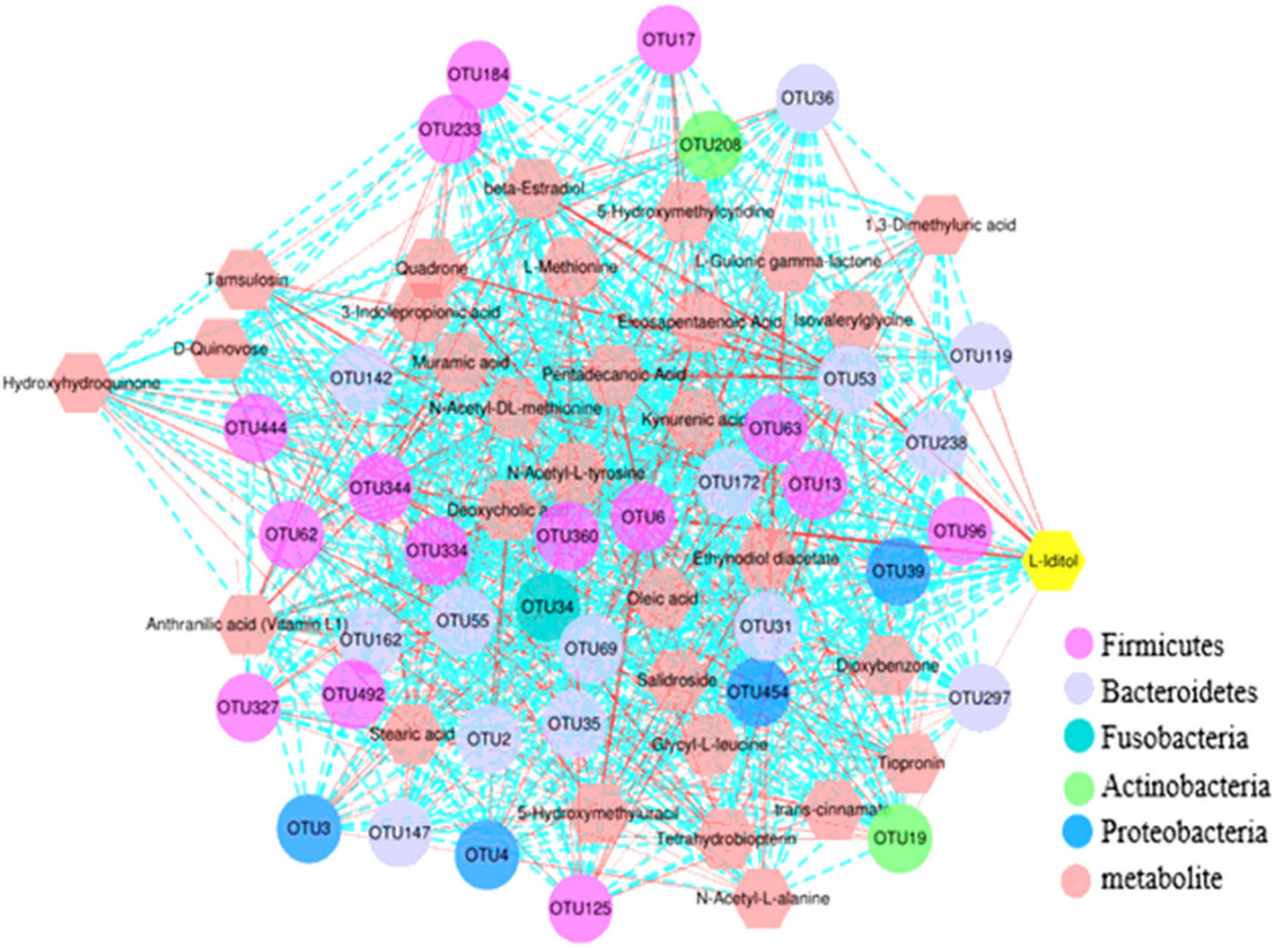
The relationships between significantly different metabolites and differential abundant OTUs in the captive and wild North China leopard. A connection indicates that the OTUs have a correlation with the metabolite,a red solid line indicates a positive correlation, a blue dashed line indicates a negative correlation, and the line thickness indicates the strength of the correlation

Spearman correlation analysis of significantly different metabolites and significantly different OTUS (microbes) was performed to obtain the relationships between metabolites and microbes (Fig. 6). In the overall network diagram, the bacteria in Firmicutes and Bacteroidetes were most closely related to metabolites, and the dominant microbes showed different positive or negative correlations to different metabolites.

In summary, the composition of the gut microbiota of captive and wild leopards was significantly different, as were the phenotypes of fecal metabolites.

## Discussion

### Gut microbiota of North China leopard and comparison with those of other carnivores

This study suggested that five major phyla, Firmicutes, Bacteroides, Actinobacteria, Proteobacteria and Saccharibacteria were observed to be different in captive and wild leopard. In addition, Fusobacteria was the major phylum in captive leopard. Firmicutes, Bacteroides, Actinobacteria and Proteobacteria were the main microbes of the captive and wild leopards, consistent with the results of studies on other carnivores, such as snow leopard [19, 20], wolves[21], Amur tiger[22], Amur leopard and cheetahs [19–26]. Fecal samples of healthy domestic cats (*Felis catus*) [27]and snow leopard (*Panthera uncia*) [19, 20, 28] featured similar phylum compositions with slightly different proportions. In our study, the relative abundance of Fusobacteria of captive and wild leopards was significantly different (*p* = 0.036), but other phyla showed no significant differences. It has been reported that bacteria of the phylum Fusobacteria are not uniformly distributed in different mammals, the contents of Cheetahs (*Acinonyx jubatus*) [26], Amur leopard (*Panthera pardus orientalis*) [29], North China leopard [24], snow leopard [19], wolves(*Canis lupus*) [23] were different.

Firmicutes was the most dominant phylum in both captive and wild leopards and it showed no significant difference between the two groups (*p* = 0.526). Many studies have reported that Firmicutes is the most predominant phylum of animals [29–31] and humans [32]. Hildebrandt reported that a high-fat diet was associated with an increase in Firmicutes and a decrease in Bacteroidetes [33]. We observed that the proportion of Firmicutes in captive North China leopard was relatively more abundant than that in wild leopards, and the proportion of Bacteroidetes in captive leopards was relatively lower, which might be related to the fact that captive animals are fed a fixed amount of food every day, unlike wild animals which hunt by themselves, resulting in greater body fat storage for the captive population.

Actinobacteria account for a large proportion of the gut microbiota of captive (13. 8%) and wild North China leopard (23.4%), and there was no significant different between the two groups (*p* = 0.473). In snow leopard [19, 20], Amur tiger [29] and North China leopard (*Panthera tigris altaica*) [24], Actinobacteria was the predominant phylum. In contrast, Wu reported that Actinobacteria constituted 0.53% in wolves[34]. Our study may indicate that Actinobacteria is the dominant phylum in felines, but there might be differences between felines and canine species, and perhaps among different mammalian species.

The relative abundance of gut probiotics such as *Lactobacillus* and *Bifidobacterium* in the wild leopard is higher than that in captive leopard. *Bifidobacterium* and *Lactobacillus* can inhibit the growth of harmful bacteria, resist infection by pathogenic bacteria such as *Staphylococcus*, *Shigella* and *Salmonella*, decompose carcinogenic substances, and improve disease resistance[35]. These abilities are largely related to the adaptation of the wild North China leopard to complex life in the wild. Moreover, the relative abundance of pathogenic bacteria such as *Shigella*, *Acinetobacter* and *Pseudomonas* in the gut microbiota of wild North China leopard is higher than that of captive leopard. The gut microbiota is a dynamically balanced ecological network, and dynamic balance and stability among the host, food and intestinal bacteria are maintained through interactions. Once the balance and stability are broken, the health of the body will be seriously threatened [36]. Additionally, the species and content of pathogenic bacteria in the gut bacteria of wild North China leopard are higher than that of captive leopard. This might indicate that the ecological network of the gut microbiota of wild leopard is in a stable state and reveals that gut microbes coevolved with the host; gut microbes were influenced by the living environment and feeding habits of animals, also affecting the physiological functions of the host [18].

### The relationships between fecal metabolites and diseases

Concerning the metabolomics, existing disease data have revealed changes in metabolic pathways and metabolites in human and animal models [37–39]. In recent years, metabolomic studies of fecal samples have found that metabolic phenotypic changes are associated with gut microbiota changes in the development of diseases, such as obesity, Crohn’s disease and colorectal cancer [40–42].Alkaline and neutral volatile metabolites such as indole and p-methylphenol are thought to be a toxin that causes blood clots, and increased bile acid is linked to an increased risk of colorectal cancer [43–45]. In our study, we found that the fecal metabolic profile of captive North China leopard was significantly different from that of wild leopards. The content of l-methionine in fecal metabolites of wild North China leopard was significantly lower than that of captive individuals. Methionine-supplementation was able to partially mitigate adverse effects caused by the higher stocking density and to improve redox status of the broilers [46], it may be a sign that the North China leopard has adapted to life in captivity. Lipid metabolism such as pentadecanoic acid, oleic acid altered in mustard airway diseases (MADs) patient [47], we found the variation of pentadecanoic acid and oleic acid on wild and captive North China leopard, but the reason for this result is not clear.

### Correlations between the gut microbiota and fecal metabolites of captive and wild North China leopard

An increasing number of studies have shown that fecal metabolites are closely related to allergic diseases, endocrine, metabolic, and gastrointestinal diseases, which can not only reflect the gut microbe ecological state but also help to diagnose and predict the occurrence and development of diseases [48–50]. In our study, we observed significant correlations between the gut microbiota and fecal metabolites using Spearman correlation analysis. Interestingly, most bacteria belonging to Firmicutes are positively correlated with metabolites; for example, Firmicutes have a strong positive correlation with N-acetyl-L-Alanine, trans-cinnamate, L-methionine, and hydroxyhydroquinone. In contrast, most bacteria belonging to Bacteroidetes are negatively correlated with metabolities; strong negative correlations were found with D-quinovose, deoxycholic acid, quadrone, and muramic acid.

Gut microbiota co-evolve with the host and adapt to different physiological and pathological states, and changes in normal microbes affect metabolic pathways. Due to the limited understanding of the gut microbiota of North China leopard, there are many unknown and future research will target diseased and healthy individuals in order to identify gut microbiota changes with potential relationships with diseases. This study provides constructive suggestions for the rational breeding of captive North China leopard and ideas for the rewilding and releasing of captive North China leopard from the perspective of the gut microbiota.

## Conclusion

Our results suggested that gut microbiota compositions and fecal metabolic phenotypes of captive and wild North China leopard were significantly different, and found that some differentially abundant gut microbes were strongly correlated with changes in fecal metabolites.

To our knowledge, this study is the first to analyze and compare the gut microbiota and fecal metabolites of captive and wild North China leopard worldwide. The results of this study are helpful for zoos to develop better management strategies for captive leopard, and provide a theoretical basis for the breeding and reintroduction of North China leopard. With the continuous deepening of gut microbiata related research, it is expected to become an effective means to guide wild animal field monitoring and disease diagnosis.

## Management suggestions

The single food of the captive North China leopard population affects the diversity of the gut microbiota; appropriate changes to the type of feed meat to be closer to the diet of the wild population would make the gut microbiota of the captive population closer to that of the wild population, which is conducive to the wild release of the captive population.

The low content of probiotics in the captive population is not conducive to better adaptation to the changeable climate. Therefore, some probiotics can be added during feeding to enrich the gut microbiota of the captive population.

Keeping the cage of the North China leopard population clean and hygienic will reduce the incidence of gut microbial infections, and once found problems can be dealt with in time.

The captive North China leopard bred by humans is not wild enough and lacks hunting skills. While the wild North China leopard lives alone, in order to survive in constant movement every day, the living area of the captive North China leopard can be set up to be larger, and living animals can be selected for feeding, thus preserving the predatory nature of the North China leopard and preparing for future wild development.

## Methods

### Fecal sample collection

#### Fecal sample collection of captive North China leopard

Three fresh fecal samples of captive North China leopard were randomly collected in Zhengzhou Zoo in Henan Province, China. Healthy leopard were randomly selected, and fecal samples were collected aseptically, immediately after defecation. The sex, age, diets and health of captive leopard used in the experiment are shown in Table 2. The fresh fecal samples were sent to the laboratory on dry ice within 24 hours of collection and stored at -80 ℃ for further microbial community analysis and metabolic analysis. We did not introduce any toxic substances that would interfere with the animal habitat. The research complied with the protocols established by the China Wildlife Conservation Association and the legal requirements of China.

**Table 2:** Sex, age, diet and health of captive leopards (A1, A4, A5) kept in Zhengzhou Zoo in Henan Province, China

Sample	Sex	Age (year)	Condition	Diet
A1	Female	1	Healthy	Duck
A4	Male	4	Healthy	Duck
A5	Male	5	Healthy	Duck

#### Fecal sample collection of wild North China leopard

Three fresh wild leopard fecal samples were collected in the Tieqiaoshan Provincial Nature Reserve in Shanxi province, China, using disposable PE gloves and sealed bags. Wild leopard frozen feces were collected aseptically immediately after they were found during winter in the reserve. The collected feces samples were transported to the laboratory with the fastest possible speed on dry ice and stored at -80℃ for the next sequencing experiment. We use leopard species-specific primer and leopard distribution areas to determine whether the leopard was North China leopard. We did not introduce any toxic substances that would interfere with the animal habitat. The research complied with the protocols established by the China Wildlife Conservation Association and the legal requirements of China.

### 16S rRNA microbial community analysis

After extracting total DNA of the sample, a primer was designed according to the conserved region, a sequencing adapter was added at the end of the primer, polymerase chain reaction (PCR) amplification was carried out, and the product was purified, quantified and homogenized to form a sequencing library; the constructed library was first subjected to library quality inspection, and the library with qualified quality inspection was sequenced with the Illumina HiSeq 2500. The original data are spliced (FLASH, version 1.2.11), the spliced sequences were quality filtered (Trimmomatic, version 0.33), and chimeras (UCHIME, version 8.1) were removed to obtain high-quality tag sequences.

### Fecal metabolic profiling

#### LC-MS analysis

LC-MS/MS analyses were performed using an UHPLC system (1290, Agilent Technologies) with a UPLC BEH Amide column (1.7 µm 2.1*100 mm, Waters) coupled to a TripleTOF 5600 (Q-TOF, AB Sciex). The mobile phase consisted of 25 mm NH4OAc and 25 mm NH4OH in water(pH = 9.75)(A), and acetonitrile (B) was carried with the elution gradient as follows:0 min, 95% B;7 min, 65% B;9 min, 40% B;9.1 min, 95% B;12 min, 95% B, which was delivered at 0.5 ml min-1. The injection volume was 3 µl. The Triple TOF mass spectrometer was used for its ability to acquire MS/MS spectra on an information-dependent basis (IDA) during an LC/MS experiment. In this mode, the acquisition software (Analyst TF 1.7, AB Sciex) continuously evaluates the full scan survey MS data as it collects and triggers the acquisition of MS/MS spectra depending on preselected criteria. In each cycle, 12 precursor ions with intensity greater than 100 were chosen for fragmentation at a collision energy (CE) of 30 V (15 MS/MS events with a product ion accumulation time of 50 msec each). ESI source conditions were set as follows: Ion source gas 1 at 60 Psi, Ion source gas 2 at 60 Psi, Curtain gas at 35 Psi, source temperature 650℃, Ion Spray Voltage Floating(ISVF) 5000 V or -4000 V in positive or negative mode, respectively [51].

### Statistical analysis

Statistical comparisons were analyzed by the Statistical Package for Social Science program (SPSS22.0, Chicago, USA). *p* < 0.05 was considered statistically significant.

## Data Availability

The datasets used and/or analysed during the current study are available from the corresponding author on reasonable request.

## Abbreviations

rRNA: Ribosomal ribonucleic acid
LC/MS: Liquid chromatography/mass spectrometer
EN: Endangered
E: Endangered
VU: Vulnerable
NT: Near Threatened
IUCN: International union for conservation of nature
CITES: Convention on International Trade in Endangered Species of Wild Fauna and Flora
DNA: Deoxyribonucleic acid
PE: Polyethylene
OPLS - DA: Orthogonal partial least squares - discrimination analysis
KEGG: Kyoto encyclopedia of genes and genomes
PCR: Polymerase Chain Reaction
UHPLC: Ultra High Performance Liquid Chromatography
NCBI: National center for biotechnology information
SRA: Short read archive
